# Invasion of *Ureaplasma diversum *in bovine spermatozoids

**DOI:** 10.1186/1756-0500-4-455

**Published:** 2011-10-27

**Authors:** Melissa Buzinhani, Maurício Yamaguti, Rosângela C Oliveira, Beatriz A Cortez, Lucas Miranda Marques, Gláucia M Machado-Santelli, Mayra EO Assumpção, Jorge Timenetsky

**Affiliations:** 1 Departamento de Microbiologia, Instituto de Ciências Biomédicas, Universidade de São Paulo, São Paulo, Brasil

**Keywords:** *Ureaplasma diversum*, bovine spermatozoid, invasion, confocal microscopy

## Abstract

**Background:**

*Ureaplasma diversum *has been associated with infertility in cows. In bulls, this mollicute colonizes the prepuce and distal portion of the urethra and may infect sperm cells. The aim of this study is to analyze *in vitro *interaction of *U. diversum *isolates and ATCC strains with bovine spermatozoids. The interactions were observed by confocal microscopy and the gentamycin internalization assay.

**Findings:**

*U. diversum *were able to adhere to and invade spermatozoids after 30 min of infection. The gentamicin resistance assay confirmed the intracellularity and survival of *U. diversum *in bovine spermatozoids.

**Conclusions:**

The intracellular nature of bovine ureaplasma identifies a new difficulty to control the reproductive of these animals.

## Background

*Ureaplasma diversum *and some mycoplasma species may cause reproductive failures in animals, but the pathogenicity of these mollicutes in such disturbances remains unclear [1]. *U. diversum *has been associated with infertility in cows and may result in severe placentitis, fetal alveolitis, abortion or birth of weak calves [2,3]. This ureaplasma can be released through milk, conjunctiva and vaginal secretions and colonize the prepuce of bulls allowing contamination of semen collected from artificial vaginas or be transmitted at sexual intercourse [3,4]. In addition, the distal urethra of bulls is highly colonized with microbes, and decontamination procedures do not sufficiently reduce the number of microorganisms in sperm, including the mollicute species [5].

Antibiotics may be added to semen before freezing in straws to prevent or reduce microbial contamination [6], yet the efficacy of this procedure has not been documented.

*Mollicutes *were initially considered surface parasites on the epithelial linings, but since 1992 in human origin mycoplasma, these bacteria have been detected inside phagocytic and non phagocytic cells [7]. Baseman et al. [8] demonstrated an intracellular localization of mycoplasmas of human origin in non phagocytic cell lines. *U. urealyticum *colonizes the human urethra and has been detected inside human spermatozoan cells [9]. The cell invasion of animal origin mycoplasmas has been recorded for some mollicutes [7-9], but this ability has not been observed for *U. diversum *in bovine spermatozoids. In this study, bovine sperm cells were infected *in vitro *with strains and field isolates of *U. diversum *and analyzed by confocal microscopy and gentamicin invasion assay.

## Results

### Analysis of labeling-*U. diversum *and motility/vigor of infected spermatozoids

The infective dose multiplicity of infection was 10^4 ^to 10^7^CCU/mL with 0.01-10 CCU/mL of ureaplasmas per spermatozoa. The viability of ureaplasmas was confirmed by production of typical ureaplasma colonies on agar plates. The average sperm motility before infection was 40% ± 14.71 and vigor 3.5. The motility and vigor after 30 min, 3 and 6 h of infection were 20% ± 12.81 and 3; 10% ± 7.35 and 2; 2.5% ± 2.75 and 1, respectively. The motility/vigor decreased by the time of incubation of infected and non infected spermatozoa.

### Microscopy of spermatozoids infected with *U. diversum*

**Figure 1:**
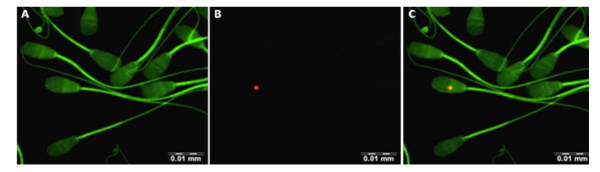
**Interaction of DiIC18-labelled *U. diversum *(isolate 94 after 30 min of infection) with FITC-labelled bovine spermatozoa *in vitro***. A: Spermatozoids in green fluorescence labeled with (FITC) B: Ureplasmas in red fluorescence labeled with (DilC) phospholipids. C: A composed of images A and B.

**Figure 2:**
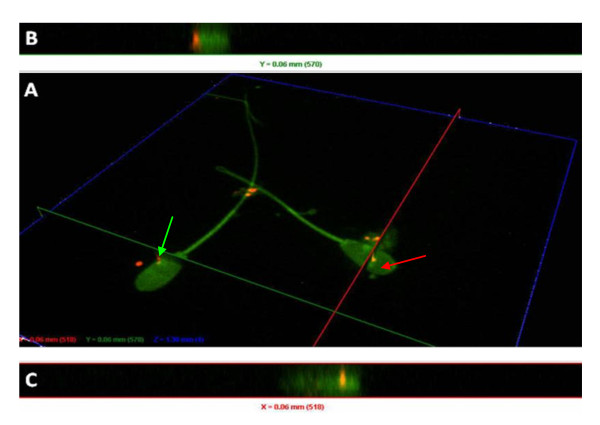
**Interaction of DiIC_18_-labelled *U. diversum *(ATCC 49782 after 3 h of infection) with FITC-labelled bovine spermatozoa *in vitro***. A: Orthogonal sectioning along the specified x-axis (red line) and y-axis (green line) with green arrows pointing to clusters of ureaplasmas attached to bovine spermatozoa and red arrows pointing to intracellular location. B: y-axis (green line) confirming the surface location of ureaplasmas (red fluorescent) in the spermatozoa. C: x-axis (red line) confirming the intercellularity of ureaplasma (red fluorescent) in the spermatozoa.

**Figure 3:**
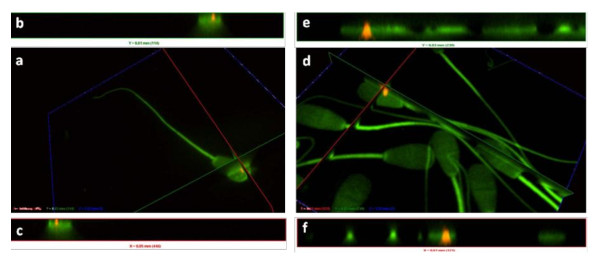
**Interaction of DiIC_18_-labelled *U. diversum *isolate 94 after 30 min (3a) and 3 h (3d) of infection with FITC-labelled bovine spermatozoa *in vitro***. a/d: Orthogonal sectioning along the specified x-axis (red line) and y-axis (green line). b/e: y-axis (green line) confirming the internal location of ureaplasmas (red fluorescent) in the spermatozoa after 30 min and 3 h of infection. c/f: x-axis (red line) confirming the internal location of ureaplasmas (red fluorescent) in the spermatozoa after 30 min and 3 h of infection.

The *U. diversum *strains and isolates were detected on the surface and inside the spermatozoids after 30 min, 3 and 6 h of infection. The ureaplasmal fluorescence (red, displayed in orange, by the overlapping of red ureaplasma and green spermatozoids actin fluorescences) was observed located on the head, middle or tail of spermatozoids, but a specific pattern of this interaction was not detected. Figure 1 also provides images obtained with the field isolates and ATCC strains of *U. diversum*. Uninfected spermatozoa showed no red fluorescence. The surface location and intracellularity of *U. diversum *(strain 49782) in spermatozoid are presented in Figure 2 by sequential sectioning of a series of focal planes. Figure 3 shows isolate 94 inside spermatozoa after 30 min (a, b, c) and 3 h (d, e, f) of infection.

### Gentamicin invasion assay

The inactivation of ureaplasma after gentamicin exposure was determined to range between 90.00 to 99.94% of the initial inoculums after 3 hours of infection (Table 1). Determination of ureaplasmal CCU/mL before and after antibiotic exposure is presented in Table 2. The obtained frequencies of CCU values ranged from 0.18 to 1.81% for studied isolates and ATCC strains from cows. The isolated TOURO1 recovered from bull sperm presented 10% frequency of invasion. The averages of invasion obtained after gentamicin exposure were not equal to the significance level of 5%. Strains 49782 and 49783 did not show differences in the average of invasion, but were lower compared with the field isolates. Strain 49782 from bull sperm presented an average significantly lower than isolate TOURO1. Average of invasion of isolate TOURO1 was significantly higher than the ATCC strains and isolates recovered from cows. The strain 49783 and the isolates from cows did not present significant differences in the average of invasion, except isolate A203. This isolate presented a higher average of invasion than others.

**Table 1:** Frequencies of *U. diversum *survival inoculated in spermatozoids after exposure to gentamicin (400μg/mL for 3 h).

Strains and isolated of *U. diversum*	Ureaplasmas inactivated by gentamicin	
	
	CCU/mL *^a^*	%
ATCC 49782	544500	99.00
ATCC 49783	5490000	99.81
A203	54000	98.18
ALVA	990000	99.00
94	5490000	99.81
3175	1000	99.94
TOURO 1	9000	90.00

*^a ^*Values CCU/mL of difference between initial inoculums and recovered after treatment with gentamicin, of 3 independent tests performed in triplicate and standard deviations.

**Table 2:** Determination of CCU/mL of *U. diversum *field isolates and ATCC strains for gentamicin invasion assay and relative frequencies of invasion.

Strains and isolates of *U. diversum*	Inoculums of tests (CCU/mL)*^a^*	Inoculums after gentamicin exposure (CCU/mL)*^a^*	Frequency of invasion (%)*^b^*
ATCC 49782	55.00 ± 0.49	0.55 ± 0.4929	1.03 ± 0.92
ATCC 49783	550.00 ± 492.95	1.00	0.18
A203	5.50 ± 0.15	0.10	1.81
ALVA	100.00	1.00	1.00
94	550.00 ± 492.95	1.00	0.18
3175	1000.00	5.50 ± 4.92	0.55 ± 0.49
TOURO 1	1.00	0.10	10

*^a ^*Values CCU/mLx10^4 ^of 3 independents tests performed in triplicate and standard deviations.

*^b ^*Percentage of ureaplasmas presenting change of colors in broth (CCU/mL) after gentamicin exposure compared to initial inoculums CCU/mL of test and standard deviations.

## Discussion

Reproductive disturbances in cattle may cause high mortality and low birth rates due the failure of conception, embryonic death and abortion. The success of embryonic development is partially predetermined by genetic characteristics of male and female gametes. However, fertilization and embryogenesis failures are both documented to be of a seminal origin [10], due to infectious agents. Mollicutes have also been isolated in reproductive tracts of bulls and cows and their presence has been related to the infertility and impairment of embryonic development Although it is believed that these bacteria remain attached to the surface of epithelial cells, studies have reported *Mycoplasma *spp. surviving inside nonphagocitic cells [8].

Winner et al. [11] reported the ability of the widespread avian pathogen *M. gallisepticum *to invade human epithelial cells and chicken embryo fibroblasts by the gentamicin invasion assay and double immunofluorescence microscopy. These techniques are considered accurate in differentiation of intracellular and extracellular location of mycoplasmas in host cells [11]. However, there are no reports of experimental infection of bovine cells with *U. diversum *by these assays. Herein the labeling *U. diversum *with DilC_18 _fluorochrome and spermatozoa with FITC-phalloidin for confocal microscopy was useful to observe the interaction of this mollicute with bovine spermatozoan cells. The DilC_18 _did not cause background fluorescence in the membranes of spermatozoids and did not affect the viability of ureaplasmas as mentioned for *M. hominis*, a human origin mollicute [12].

The infections were performed here in proportions of 0.01:1 to 10:1 CCU/mL of *U. diversum *per spermatozoid, allowing appropriate analysis. Different rates of invasion were obtained and the lower proportion of ureaplasma/spermatozoid resulted at a greater rate of invasion (10%) with isolated TOURO1. Ueno et al. [13] used the infective dose multiplicity of 1-7 *M. genitalium *per EM42 or HeLa cell and also obtained different rates of invasion.

The pool of semen from the three healthy bulls helped to standardize the study mainly for storage and the evaluation of motility and vigor of spermatozoids. The methodologies used are in accordance with Zuccari et al. [14] who concluded that the passage through a column of Percoll^® ^is effective for selecting a higher population of motile spermatozoids with intact plasmatic and acrosomal membranes.

In present study, the motility and vigor of bovine spermatozoids infected with ureaplamas did not show significant difference compared to non infected spermatozoids, in the different time-intervals of incubation. This shows that in monitoring routine procedures, the infected spermatozoids are not distinguished when selected for fertilization. Previous studies reported that human spermatozoids infected with ureaplasmas presented high motility as in uninfected cells [15,16]. Núñes-Calonge et al. [17] reported that 2 h of contact with ureaplasmas presented as high a motility, but after 4 h of contact the motility decreased compared to non infected. The infected motile spermatozoa may access and fertilize the oocytes by fertilization *in vivo *or be selected for artificial insemination [9], however, may damaged paternal DNA and impair the embryonic development [16].

The use of fluorochromes to label ureaplasma and spermatozoids allowed distinguishing these cells when coinfected and observed through confocal microscopy in contrast as described in human spermatozoa infected with mycoplamas [12,17]. Knox et al. [9] used indirect immunofluorescent methodology and showed the adherence of *U. urealyticum *and *U. parvum *on the acrosomal region, midpiece and tail of human spermatozoids. Sylla et al. [18] otherwise related a specific pattern of interaction for *M. mycoides *ssp. *mycoides *LC in the acrosomal region and tail of bovine spermatozoa. Reichart et al. [16] used electronic microscopy and detected *U. urealyticum *within the cytoplasmatic space in human and ram spermatic cells. The ability of *U. diversum *to invade bovine spermatozoids points to a new feature for better controlling the reproductive failures of these animals.

The differentiation between extracellular and intracellular ureaplasmas was performed by planes Z in a DSU system for confocality, which allowed the location of bacterial cells relative to the spermatozoids to be identified.

The detected intracellularity of *U. diversum *did not determine the integration of ureaplasma with spermatozoa DNA as questioned by Bielanski et al. [19]. However, Reichart et al. [16] concluded that *U. urealyticum *has a direct deleterious effect on the DNA and nuclear chromatin of human and ram spermatozoid. According to Cunha et al. [20], *U. urealyticum *resulted in clastogenic effects on human chromosomes and on the mitotic process itself, and the clastogenic effects varied with the *U. urealyticum *serotypes evaluated.

The invasion and survival of *U. diversum *inside bull spermatozoids were confirmed and measured by gentamicin assay as described by Yavlovich et al. [21]. The low-passage isolate (TOURO1) recovered from bull semen showed to be more invasive than the high-passage strain 49782, also recovered from bull semen. In fact, Thomas et al. [22] reported that the number of passages *in vitro *of *M. bovis *resulted in different invasion rates in cell cultures. The average rate of invasion of TOURO1 in bovine sperm was significantly higher than the isolates from cows. This may indicate that *U. diversum *recovered from bull semen may present a higher ability to invade bovine spermatozoids than the isolates recovered from cows. The binding affinity to sulfogalactoglycerolipid in germ membranes may vary among isolates of *U. diversum *and may influence their pathogenicity as suggested by Knox et al. [9]. Variations in the affinity of *Mollicute *receptors may interfere with their attachment to the host cells and may justify the variation in the rate of invasion. These variations explain the significantly higher rate of invasion of isolate A203 compared with the other isolates recovered from the same anatomic site.

The observations of *U. diversum *adherence and internalization in bull spermatozoids at 30 min of infection and the pattern of invasion did not change for the entire studied period of 6 h. Díaz-Garcia et al. [23] described the maximum adherence of *M. hominis *in human sperm after 10 min post-infection probably due to the rapid saturation of membrane receptors of the spermatozoa by excessive toxic metabolic compounds released by mollicutes [24]. The ammonia released from ureaplamas may explain in part the results obtained throughout the studied infection.

The findings in the present study warn of a need for additional care to control the quality of bull semen for reproductive techniques [25]. The regular decontamination of semen was not sufficient to eliminate microorganisms such as *M. bovis*, *M. bovigenitalium *[19] and *M. pulmonis *[26] even when antibiotics were added. Knox et al [9], detected that the standard procedures for fertilization in humans did not always remove *M. hominis *from spermatozoa. Therefore, the intracellular location of mollicutes explains the failure of antibiotics to control these bacteria.

The association of gentamycin, tylosin and lincospectin is more effective than other antibiotics in inhibiting many opportunistic microorganisms including Mollicutes in bovine semen [6,27]. The Brazilian Ministry of Agriculture, Livestock, and Food Supply [28] requires the use of gentamycin (250 μg), tylosin (50 μg), lincomycin-spectinomycin (150/300 μg) or penicillin (500 UI), streptomycin (500 UI) in semen. However, Marques et al. [29] isolated *U. diversum *in 37.14% of frozen bovine semen straws from Artificial Insemination Centers in Brazil that supply antibiotic dosages complying with national hygienic guidelines [28].

## Conclusions

The infection *in vitro *of bovine spermatozoids with *U. diversum *provides conclusive evidence of the ability of this mollicute to enter and survive inside reproductive bovine cells making the control of these bacteria more difficult. The intracellular nature of bovine ureaplasma identifies a new difficulty to control the reproductive of these animals.

## Methods

### Strains and isolates of *U. diversum *and culture conditions

*U. diversum *ATCC 49782 and 49783, and three isolates from vaginal mucus (ALVA, 3175, A203) and one isolate from bull semen (TOURO1) were studied. The microorganisms were previously stored at -70°C and then cultured at 37°C in aerobic atmosphere in Ureaplasma Broth Medium supplemented with CRML^® ^[30]. The ureaplasmal growth was expanded from 2 mL to 150 mL of broth, and confirmed by the alkaline shift and production of small dark brown colonies in solid medium [30].

### Labelling *U. diversum *cells

The ureaplasmal growth was harvested from broth cultures by centrifugation at 15,000*xg *for 50 min at 4°C. The cell pellets were homogenized in 900μl of fresh broth and mixed with 100μl of 1:50 DilC_18 _solution (Vybrant™ Dil cell-labeling, solution-Dil, V-22885, Molecular Probe, Eugene, Oregon, USA) and incubated at 37°C for 50 min. These suspensions were centrifuged at 20,600*xg *for 40 min at 4°C and washed in 1 mL PBS 2× (pH 6.5). The fluorochrome labeled ureaplasma were centrifuged again in the same conditions and homogenized in 1.3 mL Sp-TALP [31].

### Suspension of motile spermatozoids

Ejaculates from three healthy bulls were pooled and diluted with Bovimix (Nutricell^® ^Nutrientes Celulares, São Paulo, Brazil) without antibiotics and frozen at -80 °C in aliquots of 250μl. The semen was previously tested for mollicute contamination by PCR [32]. The frozen-thawed semen was layered in microtubes with a Percoll gradient (1:1 [vol/vol] mixture of 90%/45%) and centrifuged at 9,000*xg *for 5 min [33]. The spermatozoid sediments with actively motile cells were washed in 1 mL Sp-TALP by centrifugation at 6,000*xg *for 2 min, homogenized in Sp-TALP and diluted to 10^6 ^spermatozoids/100μl Sp-TALP and evaluated for motility/vigor.

### Infection of spermatozoids with *U. diversum*

The experimental infection of spermatozoids was performed as described by Díaz-Garcia et al. [12], with modifications. In microtubes, 100μl of DiIC_18_-labelled *U. diversum *cells were added to 100μl of a spermatozoid suspension in microtubes. The ureaplasmal infected spermatozoids and the control of non infected spermatozoid suspensions were incubated at 37°C in 5% CO_2 _for 30 min, 3 and 6 h. These tests were performed in duplicate for each time of incubation. The infective dose multiplicity was determined as described by Taylor-Robinson [34]. An aliquot of bacterial suspension was 10-fold diluted in Ureaplasma broth for determination of CCU/mL. The viability of bacteria at different periods of incubation was confirmed on agar plates. Aliquots of infected and non infected spermatozoid suspensions were taken at different time-intervals for analysis of sperm motility/vigor. The motility was determined subjectively in percentage (%) and the vigor on a scale of 0 to 5, by microscopy (400×). For confocal microscopy the spermatozoid cells were initially fixed with paraformaldehyde 3.7% for 20 min at room temperature, washed twice in PBS by centrifugation (6,000 *× g *for 2 min) and homogenized in 100μl of PBS and stored at 4°C.

### Confocal Microscopy

Internalization of *U. diversum *into bovine spermatozoids was distinguished by fluorescence differences of fluorochromes exposed to the light filters of a confocal microscope. One-hundred fixed spermatozoid suspensions were centrifuged onto glass slides (76 × 26 mm/3 × 1 inch) by a cytological centrifuge. The smears were permeabilized with Triton X-100 0.5% for 10 min, washed with PBS and labeled with 10μl (1:200) of FITC-phalloidin (Sigma^®^) for 40 min at room temperature. Next, the smears were washed twice in PBS, air-dried and mounted with a 24 × 50 mm coverslip and Vectashield mounting fluid (Vector Laboratories, Burlingame, CA, USA). The slides with infected and non infected spermatozoids were observed by a Motorized Inverted System Microscope IX81 Olympus^®^), coupled with an MT20 illumination system and a 60× oil immersion objective. The plane Z-axis control was determined at 0.5μm.

### Gentamicin invasion assay

Labeled infected and non infected paired spermatozoid suspensions were incubated at 37°C with 5% CO2 for 3 h. Next, these suspensions were washed in PBS and centrifuged at 6,000*xg *for 2 min. The obtained pellet of spermatozoids was homogenized with 200μl Sp-TALP with 400μg/ml gentamicin and incubated for 3 h [21]. The antibiotic exposed suspension was centrifuged and spermatozoid sediment was homogenized with 200μl of Ureaplasma medium and 10-fold diluted with the same broth, in triplicate, for determination of CCU/mL [34]. Each gentamicin resistance assay was repeated three times. Invasion frequencies were calculated as the difference of CCU/mL of ureaplasma suspension samples before and after gentamicin exposure. The differences were calculated by Student's *t *test. Differences with *P = *0.05 were considered significant.

## Competing interests

The authors declare that they have no competing interests.

## Authors' contributions

MB and JT: all tests realized in this study. BAC and GMMS: confocal analysis. MY, RCO, LMM: bacteria isolation and infection experiment. MEOA: motile spermatozoids acquisition. All authors read and approved the final manuscript.
